# Intravesical Botulinum Toxin Type A for an Overactive Bladder: A Single-Center Audit

**DOI:** 10.7759/cureus.98526

**Published:** 2025-12-05

**Authors:** Philip Abolanle, Thomas M Richards, Maike Eylert, Rebecca Saunders, Paulette Hussain, Coral Seymour

**Affiliations:** 1 Urology Department, Royal Gwent Hospital, Aneurin Bevan University Health Board, Newport, GBR; 2 Functional Urology Nursing, Urology Department, Royal Gwent Hospital, Aneurin Bevan University Health Board, Newport, GBR

**Keywords:** botulinum toxin injection, detrusor overactivity, intravesical treatment, neurogenic bladder dysfunction, overactive bladder, refractory overactive bladder, urine incontinence

## Abstract

Background

Intravesical administration of botulinum toxin type A (BoNT-A) is an established third-line intervention for patients with refractory overactive bladder (OAB). With BoNT-A therapy now widely adopted, ongoing audits remain important to capture real-world outcomes across different patient groups. This retrospective audit assessed the clinical outcomes of BoNT-A therapy in a heterogeneous patient population treated at a university-affiliated teaching hospital.

Methodology

We conducted a retrospective audit of 193 patients who received intravesical BoNT-A injections for OAB between January and December 2024. Patients underwent urodynamic testing to confirm diagnosis, and all cases were discussed in multidisciplinary team meetings where approval was given for BoNT-A treatment. Demographic data, treatment parameters, efficacy outcomes, adverse events, and catheterization requirements were systematically analyzed. Statistical analyses included chi-square tests and Fisher’s exact tests to evaluate associations between variables.

Results

The cohort included 164 (85%) females and 29 (15%) males, with a median age of 62 years. The primary indications for BoNT-A therapy were idiopathic detrusor overactivity (IDO, n = 149, 77.2%), neurogenic detrusor overactivity (NDO, n = 41, 21.2%), and sensory urgency (n = 3, 1.6%). Treatment success was observed in 82.4% (n = 159) of patients, with partial improvement in 4.1% (n = 8) and treatment failure in 11.9% (n = 23). The overall urinary tract infection (UTI) rate was 4.7% (n = 9), with diabetic patients exhibiting a three-fold increased risk (11.5% vs. 3.6%), but this did not reach statistical significance (χ² = 3.195, df = 1, p = 0.074; Fisher’s p = 0.105). Post-treatment catheterization was required in 29.5% (n = 57) of cases, predominantly among patients with NDO (n = 22, 53.7% vs. IDO, n=37, 25%). First-time BoNT-A administration occurred in 21.7% (n = 42) of patients, while the majority (n = 151, 78.3%) received repeat injections during the study period, with a mean of seven treatment cycles. The median duration of effect following intravesical BoNT‑A was six months (interquartile range = 5). The mean duration was 6.7 ± 4.0 months.

Conclusions

Intravesical BoNT-A demonstrates robust efficacy and an acceptable safety profile in the management of refractory OAB, with sustained therapeutic benefits necessitating repeat administration. Diabetic status may predispose patients to an increased risk of post-procedural UTIs, while those with NDO are more likely to require catheterization. These findings support BoNT-A as a valuable therapeutic option in the stepwise management of OAB, particularly when combined with appropriate patient selection and counseling.

## Introduction

Overactive bladder (OAB) syndrome poses a substantial global healthcare challenge, affecting millions of individuals and significantly impacting quality of life. According to the International Continence Society (ICS), OAB is defined as urinary urgency, typically accompanied by increased frequency and nocturia, with or without urge incontinence, in the absence of urinary tract infection (UTI) or other identifiable pathology [1]. The underlying pathophysiology is commonly attributed to detrusor overactivity (DO), characterized by involuntary detrusor contractions, either spontaneous or provoked, during the bladder filling phase on urodynamic assessment [1,2].

DO is classified as neurogenic (NDO) when associated with neurological conditions such as spinal cord injury, multiple sclerosis, Parkinson’s disease, or cerebrovascular accidents, and idiopathic (IDO) when no clear neurological cause is identified. Epidemiological studies suggest that more than 90% of women with OAB symptoms have IDO [2]. The condition significantly impacts quality of life, with patients experiencing social embarrassment, sleep disturbance, reduced productivity, and psychological distress.

Management of OAB typically follows a stepwise therapeutic algorithm. First-line interventions include behavioural strategies such as bladder retraining, pelvic floor muscle exercises, and fluid intake optimization. Pharmacologic therapy constitutes the second-line approach, primarily involving antimuscarinic agents or beta-3 adrenergic agonists, with emerging evidence supporting combination regimens to improve efficacy [3]. Nonetheless, adverse effects and limited tolerability contribute to suboptimal outcomes, with an estimated 30-40% of patients exhibiting inadequate response to pharmacotherapy [4].

For patients unresponsive to conservative measures and pharmacologic therapy, third-line interventions include intravesical BoNT-A injections, posterior tibial nerve stimulation, and sacral neuromodulation. Historically, surgical procedures such as bladder augmentation and urinary diversion have been reserved as last-resort options due to their invasive nature and associated morbidity [4].

Botulinum toxin (BoNT), initially recognized as a causative agent of botulism following its discovery by Emile van Ermengem in 1895 [5], has been transformed into an effective therapeutic agent across multiple medical specialties. The neurotoxin, produced by the anaerobic bacterium *Clostridium botulinum*, exists in seven antigenically distinct serotypes (A-G), with types A and B commercially available for clinical use. BoNT-A exerts its therapeutic effect by inhibiting calcium-dependent acetylcholine release at the presynaptic neuromuscular junction, leading to temporary chemodenervation and muscle paralysis [6].

In the treatment of OAB, intravesical BoNT-A injection induces transient paresis of the bladder wall, effectively suppressing involuntary detrusor contractions while maintaining voluntary voiding function in the majority of patients, although a proportion require intermittent catheterization. The first clinical applications in neurogenic bladder were reported by Schurch et al. in 2000, demonstrating complete continence restoration in 17 of 19 patients with spinal cord injury, with effects lasting at least nine months [7]. Subsequent randomized controlled trials and systematic reviews have established robust evidence for efficacy in both neurogenic and idiopathic OAB [8-10].

Despite growing adoption of BoNT-A therapy for OAB, questions remain regarding optimal patient selection, predictors of treatment success, management of adverse events, and long-term outcomes in routine clinical practice. This audit aimed to describe real‑world outcomes of intravesical BoNT‑A therapy for refractory OAB in a single‑center cohort of 193 patients, focusing on treatment efficacy, safety, and factors influencing therapeutic response.

This study has been previously presented as a poster at the ASiT Innovation Summit on November 7th, 2025.

## Materials and methods

Study design and patient selection

A retrospective audit was performed on all patients who received intravesical BoNT-A injections for OAB at our institution between January and December 2024. All patients had previously failed conservative interventions and at least one pharmacologic agent, including antimuscarinic drugs or beta-3 adrenergic agonists. The diagnosis of DO was confirmed via urodynamic studies before the initiation of BoNT-A therapy. Patients were classified into the following three diagnostic categories based on urodynamic findings and clinical presentation: IDO, NDO, and sensory urgency. All cases were discussed at our multidisciplinary team (MDT) meeting to obtain treatment approval. The MDT consisted of relevant specialists as recommended by the National Institute for Health and Care Excellence (NICE) guidelines [3]. Approval criteria included review of the patients’ clinical presentation and urodynamic findings, alongside confirmation that first‑ and second‑line treatments had been reasonably explored. Demographic data collected included age, gender, diabetic status, and relevant medical history.

Pre-procedural patient preparation

All patients received standardized pre‑procedural counseling, supported by written information leaflets and a dedicated clinic appointment to learn intermittent self-catheterization (ISC) and discuss treatment expectations. Informed consent was obtained and documented before BoNT‑A administration. Patients unable or unwilling to perform ISC were advised of the alternative option of indwelling catheterization; those declining both options were not given the treatment, in line with NICE guidelines [3].

Patients maintaining normal voiding patterns post-injection were advised that ISC was not necessary. Those attempting ISC who consistently obtained residual volumes of less than 200 mL were counseled that regular catheterization was not required. Intermittent catheterization was defined as the occasional need to void using a catheter, rather than routine or continuous use. A three-day bladder diary was often completed by patients at baseline to document voiding frequency, urgency episodes, and incontinence events.

Injection technique

In 95% of cases, injections were performed under local anesthesia with flexible cystoscopy; 5% (n = 10) required general anesthesia with rigid cystoscopy, following established technical protocols [11,12]. Ten injections were distributed across the anterior, lateral, and posterior bladder walls, avoiding areas of abnormality such as diverticula, prior resection sites, and the dome. The trigone was spared in all cases, with no inadvertent injections or subsequent vesicoureteral reflux reported.

All patients were provided with discharge instructions, including safety‑netting advice for hematuria and UTI, encouragement of good hydration, and contact details for the emergency assessment unit. No specific activity restrictions were required, and pain was generally minimal.

Antibiotic prophylaxis

Antibiotic prophylaxis was administered to all patients to minimize the risk of post-procedural UTI according to institutional guidelines, with the choice between single‑dose and short‑course regimens guided by patient‑specific risk profile. The majority (n = 150, 77.7%) received a single dose of ofloxacin 400 mg orally pre-procedure. Short-course regimens (3-5 days) were prescribed in 21.8% (n = 42) of cases.

Outcome measures

Treatment outcomes were assessed by patient‑reported improvement, categorized as success, partial benefit, or failure, and corroborated by reduction in urgency episodes, pad usage, and subjective quality of life. Patients receiving their first BoNT-A treatment were reviewed two weeks post-procedure. Those undergoing repeat injections were provided direct access to functional urology nursing support for any post-treatment concerns. Primary outcome measures included: (1) treatment success, defined as patient-reported significant improvement in OAB symptoms; (2) partial benefit, defined as modest symptom improvement; and (3) treatment failure, defined as absence of perceived benefit. Secondary outcomes comprised the incidence of UTI, requirement for catheterization (clean intermittent or indwelling), duration of therapeutic effect, and frequency of repeat injections.

UTI was diagnosed based on symptomatic presentation combined with positive urine cultures. Catheterization requirements were categorized as no catheter needed, pre-existing long-term catheter, occasional/PRN ISC, or regular ISC. Duration of treatment effect was recorded from the date of injection until symptom recurrence necessitating repeat treatment or alternative management. Patients were advised to discontinue oral therapies following BoNT‑A to assess the isolated effect. Some patients resumed their medications when the perceived effect was suboptimal or had worn off before the next appointment. This subgroup was not analyzed separately.

Statistical analysis

Descriptive statistics were used to summarize demographic and clinical variables. Categorical data were analyzed using chi-square tests or Fisher’s exact tests when expected cell counts were small. Continuous variables were reported as means with standard deviations or medians with interquartile ranges, as appropriate. A p-value <0.05 was considered statistically significant. All statistical analyses were conducted using standard analytical software.

## Results

Patient demographics

**Figure 1:**
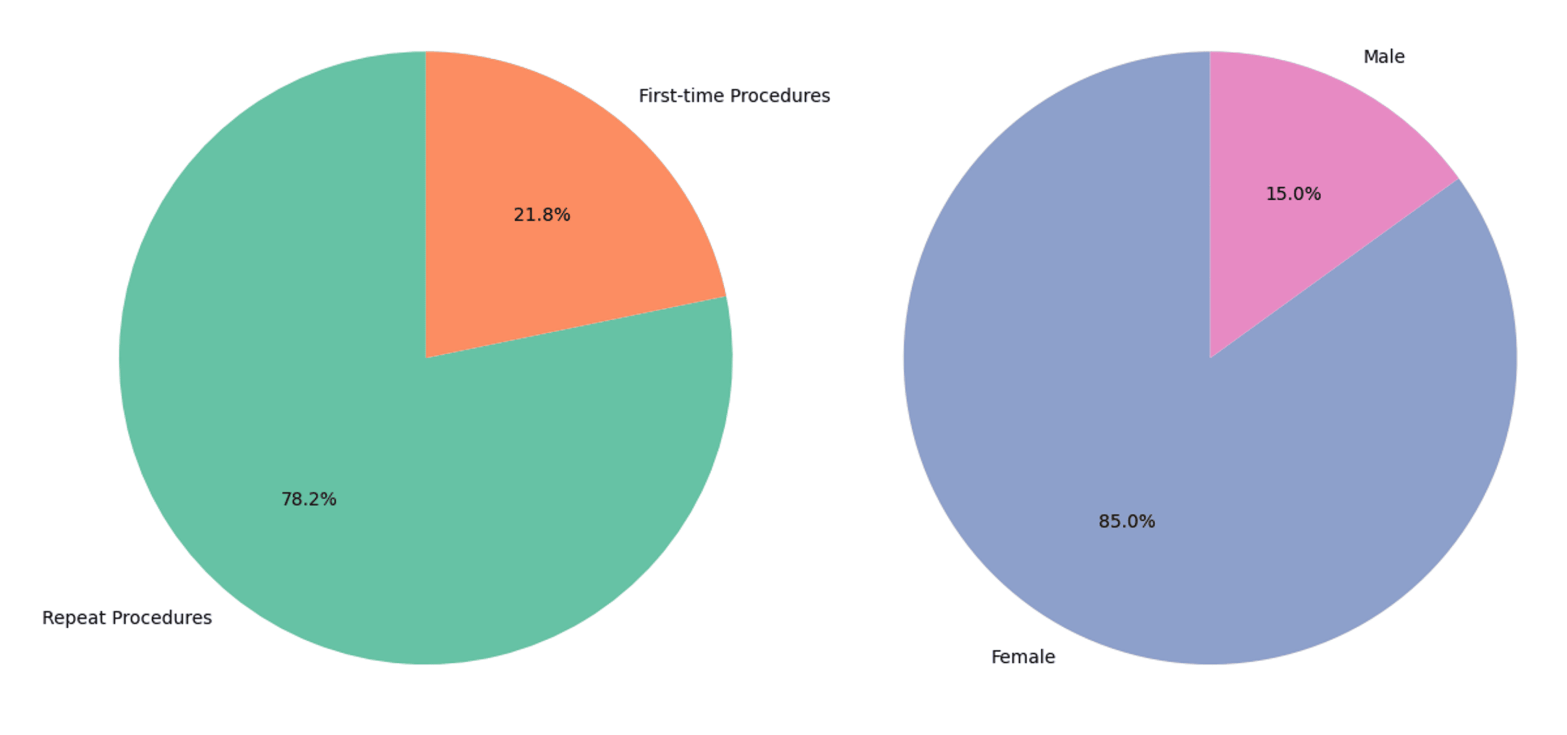
Botulinum toxin type A (BoNT-A) treatment history and gender distribution.

During the study period, 193 patients received intravesical BoNT-A injections. The cohort exhibited a marked female predominance, comprising 164 (85%) females and 29 (15%) males. Diabetes mellitus was documented in 26 (13.5%) patients, while 167 (86.5%) had no recorded history of the condition; this was the only systematically captured comorbidity. The majority of treatments (78.3%, n = 151) were repeat procedures, with 42 (21.7%) patients undergoing BoNT-A for the first time (Figure 1). Among those receiving repeat injections, the mean number of prior treatment cycles was seven, reflecting sustained long-term utilization of BoNT-A therapy.

Clinical indications

IDO was the predominant indication for BoNT-A treatment, accounting for 149 (77.2%) patients, followed by NDO in 41 (21.2%) patients, and sensory urgency in three (1.6%) patients. This distribution aligns with the predominantly idiopathic etiology of OAB observed in the general population. The predominant neurological diagnoses were multiple sclerosis, spinal cord injury, and Parkinson’s disease. Prior third‑line therapies, such as neuromodulation, were not routinely encountered in our cohort.

Regarding concomitant bladder medications at the time of BoNT-A injection, 120 (62.2%) patients were not receiving any pharmacological therapy, having discontinued antimuscarinic or beta-3 agonist medications due to inefficacy or intolerable side effects. Among those receiving concurrent therapy, 31 (16.1%) patients used beta-3 agonists, 22 (11.4%) used anticholinergics, 19 (9.8%) used combination therapy, and one (0.5%) was taking duloxetine for mixed urinary symptoms.

Dosing and dose escalation

The vast majority of patients (96.9%, n = 187) received consistent dosing across treatment cycles without dose escalation. Only six (3.1%) patients had their BoNT-A dose increased during this period, suggesting a stable long-term therapeutic response in most patients.

Treatment efficacy

Among first-time recipients (n = 42), 34 (81%) patients reported noticeable improvement within two weeks post-injection, five reported no change, and three were lost to follow-up. Among repeat treatment patients (n = 151), 11 (7%) experienced reduced efficacy from their most recent injection and returned early for re-treatment. Reduced efficacy was defined as patient‑reported perception of diminished benefit compared with their prior injection cycle, rather than the expected waning of effect at 6-12 months. This was managed by repeating treatment at the same dose before considering escalation. The duration of treatment effect varied widely, ranging from no response (0 months) to sustained benefit exceeding 18 months. The most frequently reported duration was 6-12 months, consistent with the known pharmacodynamics of BoNT-A. These findings underscore the importance of repeat injections to maintain therapeutic benefit in the majority of patients.

**Figure 2:**
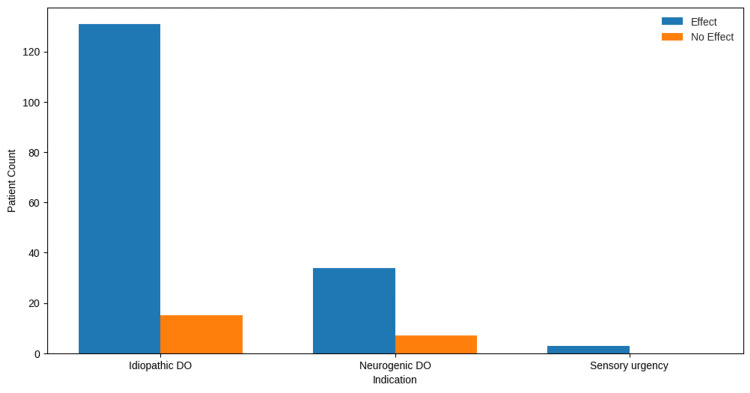
Treatment effect by indication type. DO = detrusor overactivity

When stratified by diagnostic category (Figure 2), treatment response did not significantly differ between IDO, NDO, and sensory urgency groups (χ² = 1.84, df = 2, p = 0.3976). However, the limited sample size for the sensory urgency subgroup (n = 3) restricts the statistical power to detect meaningful differences within this cohort.

Urinary tract infections

Post-procedural UTI occurred in nine (4.7%) patients, with a higher incidence observed among diabetic patients (n = 3, 11.5%, 95% confidence interval (CI) = 4.0-29.0%) compared to non-diabetics (n = 6, 3.6%, 95% CI = 1.7-7.7%), indicating a three-fold increased risk. Although statistical significance was not reached (χ² = 3.195, df = 1, p = 0.074; Fisher’s exact p = 0.105), the trend suggests a clinically relevant association. The limited number of diabetic patients (n = 26) may have constrained statistical power. CIs were wide due to small numbers, and these findings should be considered hypothesis‑generating. UTI occurred in 7/150 (4.7%) patients receiving single‑dose prophylaxis and 2/42 (4.8%) patients receiving short‑course prophylaxis, with comparable rates between regimens.

Catheterization requirements

Post-procedural catheterization was not required in 58.5% (n = 113) of patients, while 57 (29.5%) developed new catheterization needs (either intermittent or regular ISC). Among patients with IDO, 112 (75%) avoided catheterization, and higher BoNT-A doses (≥200 units) were not significantly associated with increased catheter use (χ² = 13.51, df = 12, p = 0.333). In contrast, over half of patients with NDO required new catheterization (n = 22, 53.7%). Statistical analysis did not demonstrate a significant association between BoNT‑A dose and catheterization within this subgroup (χ² = 16.24, df = 20, p = 0.702). These findings are consistent with the possibility that underlying neurogenic etiology, rather than BoNT‑A dosage, may contribute to post‑treatment catheterization risk, although this should be interpreted with caution.

## Discussion

This audit of 193 consecutive patients receiving intravesical BoNT-A therapy for refractory OAB demonstrates robust efficacy and acceptable safety in real-world clinical practice. The overall success rate of 82.4% aligns closely with pooled data from high-level randomized controlled trials, which report success rates of 55-85% depending on outcome definitions [9,10]. Our findings reinforce the established role of BoNT-A as a valuable third-line treatment option for patients who have failed conservative and pharmacological management.

Efficacy and duration of effect

In our cohort, the median duration of therapeutic benefit following BoNT-A injection was 6-12 months, necessitating repeat administration to sustain symptom control. This aligns with BoNT-A’s pharmacodynamic profile, wherein chemodenervation gradually diminishes as neuromuscular junctions regenerate and acetylcholine transmission resumes [6,7]. The finding that 78.3% of patients received repeat treatments, with an average of seven injection cycles, reflects both the enduring efficacy and high patient acceptance of this intervention over time. Recent evidence from Manso et al. (2024) corroborates our findings, reporting that 60.3% of women required repeat BoNT-A [13]. Importantly, they found no objective evidence of diminishing efficacy with repeated treatments, despite subjective patient perceptions of reduced benefit.

The predictors of treatment success identified in the literature include lower baseline post-void residual volume, higher maximum flow rate, and larger voided volumes [14,15]. While our audit did not systematically capture these urodynamic parameters, future prospective studies incorporating detailed baseline urodynamics may enable more precise patient selection and outcome prediction.

Safety profile and adverse events

The post-procedural UTI rate of 4.7% in our cohort compares favorably to the 7-21% range reported in placebo-controlled trials [9]. This low infection rate likely reflects our systematic use of antibiotic prophylaxis, which was administered to all patients. The three-fold increased UTI risk observed in diabetic patients (11.5% vs. 3.6%), while not reaching statistical significance (p = 0.074), represents a clinically meaningful finding that warrants consideration in treatment planning. While single-dose regimens minimize antibiotic exposure and resistance development, short courses (3-5 days) may provide additional protection in high-risk populations. Our data showed that both single-dose and short-course regimens were represented among patients who developed UTI, preventing definitive conclusions regarding the superiority of either approach. Prospective randomized studies are needed to establish evidence-based prophylaxis protocols.

Catheterization requirements

The 29.5% rate of new catheterization following BoNT-A injection represents a crucial consideration in patient counseling and informed consent. However, this aggregate figure conceals notable variation between diagnostic groups. Patients with IDO had significantly lower catheterization rates (25%) compared to those with NDO (53.7%), reflecting the preserved neurological coordination of voiding in idiopathic cases. Our finding that higher BoNT-A doses (≥200 units) did not significantly increase catheterization risk in IDO patients (p = 0.158) contrasts with some published reports [8,16] and warrants further investigation. The absence of a dose-response relationship may reflect preserved baseline detrusor contractility in IDO, enabling continued voiding despite moderate chemodenervation. In contrast, the elevated catheterization rate among NDO patients likely reflects the multifactorial pathophysiology of neurogenic bladder, including detrusor-sphincter dyssynergia, impaired sensation, and reduced contractility. Ribeiro et al. (2023) identified high baseline post-void residual and BoNT-A doses >100 units as predictors of ISC requirement in men, while prior bladder outlet surgery was protective [15]. These findings highlight the need for individualized dose selection and comprehensive pre-treatment urodynamic assessment, particularly in neurogenic populations. A key finding from our audit is that most patients requiring catheterization post-BoNT-A injection had received pre-procedural training in ISC. This structured approach, educating all patients in the ISC technique while permitting those with preserved voiding to avoid routine catheterization, offers a pragmatic balance between patient autonomy and procedural safety. It aligns with current best practice guidelines, which emphasize comprehensive patient education regarding the potential need for temporary or ongoing catheterization [12,13].

Technical considerations

The overwhelming majority of procedures (95%) in our series were performed under local anesthesia using flexible cystoscopy, demonstrating excellent patient tolerance of this minimally invasive approach. The standard technique of 10 injection sites distributed across the bladder wall, sparing the trigone, represents a simplified protocol compared to some studies employing 20-40 injection sites. Recent evidence from Zdroik et al. (2024) found no significant differences in pain, efficacy, or adverse events between 10-site and 20-site injection protocols [16], supporting our institutional practice and potentially reducing procedure time and patient discomfort.

Cost and quality outcomes

Although cost-effectiveness was not directly assessed in this audit, existing economic evaluations consistently support the value of BoNT-A therapy in managing refractory OAB. Studies such as those by Freemantle et al. (2016) demonstrated that onabotulinumtoxinA offers greater health benefits at lower costs compared to best supportive care, with a high probability of cost-effectiveness within UK thresholds [17]. Additional European modeling has shown long-term budget savings, and comparisons with anticholinergic therapy suggest BoNT-A achieves sustained efficacy at reduced cost beyond six months [18,19]. These findings, coupled with patient preference for procedural over daily pharmacological interventions, reinforce the economic viability of BoNT-A.

While formal quality of life metrics were not captured in this retrospective review, the high rate of repeat treatments (78.3%) and long-term adherence suggest meaningful patient-perceived benefit. Prospective studies, including Licow et al. (2023), have reported significant improvements in symptom burden and all domains of disease-specific quality of life following BoNT-A therapy. These benefits extend to psychosocial functioning, sleep, and productivity, helping explain the high satisfaction and continued uptake observed despite the need for periodic re-injections and potential catheterization [20].

Limitations

Outcome assessment relied primarily on subjective patient-reported improvement rather than standardized validated instruments or objective urodynamic parameters. While patient perception represents the ultimate clinically meaningful endpoint, incorporation of validated questionnaires (International Consultation on Incontinence Questionnaire-Overactive Bladder (ICIQ-OAB), Overactive Bladder Questionnaire (OAB-q)) and repeat urodynamic studies would strengthen future analyses. Baseline post-void residual volume was not analyzed in detail, as the study’s focus was on treatment outcomes rather than predictors of catheterization. Elevated post-void residual volume in NDO patients may contribute to catheterization risk and should be considered a potential confounder.

Additionally, variability in the definition of urinary retention and criteria for initiating catheterization across the study period reflects evolving institutional practices. As noted by Stavrou et al. [21], such heterogeneity complicates cross-study comparisons and patient counseling. Standardizing retention definitions and catheterization thresholds would enhance the robustness of future research.

Future directions

Future research should focus on identifying additional predictors of BoNT-A treatment success through prospective studies with comprehensive baseline urodynamics. Optimizing antibiotic prophylaxis, particularly for high-risk groups such as diabetics, remains a key area for investigation. The subjective perception of diminishing efficacy with repeated injections warrants mechanistic exploration to guide dosing and counseling. Future prospective studies should incorporate validated quality of life instruments such as the OAB‑q and ICIQ‑OAB to complement clinical outcomes and provide a more comprehensive assessment of patient‑perceived benefit. Comparative trials between BoNT-A and sacral neuromodulation could inform third-line treatment decisions. Emerging applications in pediatric urology, post-augmentation cystoplasty, and bladder pain syndrome also merit further investigation, alongside refinements in injection technique to improve outcomes and reduce adverse events [22,23].

Clinical implications

This audit supports several key clinical recommendations: BoNT-A is a highly effective third-line therapy for refractory OAB, with success rates exceeding 80% in well-selected patients. Pre-procedural counseling should address differential catheterization risks, particularly in NDO patients, who face a ~50% likelihood of requiring ISC. Diabetic patients warrant closer monitoring for post-injection UTI and may benefit from tailored prophylaxis strategies. Clear communication regarding the expected duration of benefit (6-12 months) and the need for repeat injections is essential. Universal ISC training before treatment, coupled with individualized post-injection protocols, offers a pragmatic and patient-centered approach to managing retention risk.

## Conclusions

This audit reinforces intravesical BoNT-A as a safe and effective third-line therapy for refractory OAB, with over 80% treatment success and sustained symptom relief lasting 6-12 months. UTI rates were low overall, and catheterization requirements were acceptable for many patients, though risks were clearly higher in neurogenic cases (53.7%) and in diabetic patients, where a trend toward increased UTI was observed. These diagnosis‑specific differences highlight the importance of individualized counseling and shared decision‑making. High repeat treatment rates reflect durable efficacy and patient acceptance. As OAB management evolves, BoNT-A remains a valuable option between pharmacotherapy and surgery.

## References

[1] Abrams P, Andersson KE, Birder L (2010). Fourth International Consultation on Incontinence Recommendations of the International Scientific Committee: evaluation and treatment of urinary incontinence, pelvic organ prolapse, and fecal incontinence. Neurourol Urodyn.

[2] Orasanu B, Mahajan ST (2013). The use of botulinum toxin for the treatment of overactive bladder syndrome. Indian J Urol.

[3] (2025). National Institute for Health and Care Excellence (NICE). Urinary incontinence and pelvic organ prolapse in women: management. https://www.nice.org.uk/guidance/ng123.

[4] Babin CP, Catalano NT, Yancey DM (2024). Update on overactive bladder therapeutic options. Am J Ther.

[5] van Ermengem E (1979). Classics in infectious diseases. A new anaerobic bacillus and its relation to botulism. E. van Ermengem. Originally published as "Ueber einen neuen anaëroben Bacillus und seine Beziehungen zum Botulismus" in Zeitschrift für Hygiene und Infektionskrankheiten 26: 1-56, 1897. Rev Infect Dis.

[6] Simpson LL (1980). Kinetic studies on the interaction between botulinum toxin type A and the cholinergic neuromuscular junction. J Pharmacol Exp Ther.

[7] Schurch B, Stöhrer M, Kramer G, Schmid DM, Gaul G, Hauri D (2000). Botulinum-A toxin for treating detrusor hyperreflexia in spinal cord injured patients: a new alternative to anticholinergic drugs? Preliminary results. J Urol.

[8] Mangera A, Apostolidis A, Andersson KE (2014). An updated systematic review and statistical comparison of standardised mean outcomes for the use of botulinum toxin in the management of lower urinary tract disorders. Eur Urol.

[9] Herschorn S, Gajewski J, Ethans K (2011). Efficacy of botulinum toxin A injection for neurogenic detrusor overactivity and urinary incontinence: a randomized, double-blind trial. J Urol.

[10] Wang Q, Li C, Long Y (2025). Efficiency and safety of noninvasive and intravesical therapy for adult neurogenic lower urinary tract dysfunction: a systematic review and network meta-analysis of randomized controlled trials. Drugs.

[11] Karsenty G, Baverstock R, Carlson K (2014). Technical aspects of botulinum toxin type A injection in the bladder to treat urinary incontinence: reviewing the procedure. Int J Clin Pract.

[12] Smith CP, Chancellor MB (2005). Simplified bladder botulinum-toxin delivery technique using flexible cystoscope and 10 sites of injection. J Endourol.

[13] Manso M, Soares JD, Henriques M, Botelho F, Silva C, Cruz F (2024). Efficacy, satisfaction, and compliance: insights from 15 years of botulinum toxin use for female urgency urinary incontinence. Toxins (Basel).

[14] Lee YK, Kuo HC (2025). Urodynamic predictive factors for successful treatment outcomes following intravesical botulinum toxin a injection in patients with detrusor overactivity. Biomedicines.

[15] Ribeiro L, Leung LY, Tan N (2023). Predictors for adverse events following intravesical botulinum toxin injections in men. Neurourol Urodyn.

[16] Zdroik A, El Haraki A, Smith W, Badlani G, Parker-Autry C, Matthews C (2024). Injection site number and outcomes of intradetrusor onabotulinumtoxinA for refractory overactive bladder syndrome: a randomized clinical trial. Int Urogynecol J.

[17] Freemantle N, Khalaf K, Loveman C, Stanisic S, Gultyaev D, Lister J, Drake M (2016). OnabotulinumtoxinA in the treatment of overactive bladder: a cost-effectiveness analysis versus best supportive care in England and Wales. Eur J Health Econ.

[18] Ruff L, Bagshaw E, Aracil J, Velard ME, Pardhanani G, Hepp Z (2016). Economic impact of onabotulinumtoxinA for overactive bladder with urinary incontinence in Europe. J Med Econ.

[19] Visco AG, Zyczynski H, Brubaker L (2016). Cost-effectiveness analysis of anticholinergics versus botox for urgency urinary incontinence: results from the anticholinergic versus botox comparison randomized trial. Female Pelvic Med Reconstr Surg.

[20] Licow A, Ciecwiez S, Brodowska A (2023). Quality of life in patients with overactive bladder following botulinum toxin treatment: a preliminary report. Ginekol Pol.

[21] Stavrou S, Paynter JA, Carins T, Qin KR, Brennan J (2025). Variation in the definitions of urinary retention in studies of intravesical botulinum toxin for idiopathic overactive bladder: a narrative systematic review. Neurourol Urodyn.

[22] Mohan Kunnath S, Solomon E, Mishra P, Wright AJ, Clothier J, Garriboli M (2025). Intravesical botulinum toxin injection for treating detrusor overactivity and poor compliance in posterior urethral valves-a preliminary experience. Neurourol Urodyn.

[23] Martinez L, Tubre R, Roberts R, Boone T, Griebling TL, Padmanabhan P, Khavari R (2020). Refractory bladder dysfunction: a multi-institutional experience with intravesical botulinum toxin-a injection in adult patients who underwent previous augmentation cystoplasty. Turk J Urol.

